# Breathing New Life into the Mechanisms of Platinum Resistance in Lung Adenocarcinoma

**DOI:** 10.3389/fcell.2020.00305

**Published:** 2020-05-08

**Authors:** Alvaro Gonzalez-Rajal, Jordan F. Hastings, D. Neil Watkins, David R. Croucher, Andrew Burgess

**Affiliations:** ^1^ANZAC Research Institute, Concord, NSW, Australia; ^2^The Kinghorn Cancer Centre, Garvan Institute of Medical Research, Sydney, NSW, Australia; ^3^Research Institute in Oncology and Hematology, Cancer Care Manitoba, Winnipeg, MB, Canada; ^4^Department of Internal Medicine, Rady Faculty of Health Sciences, University of Manitoba, Winnipeg, MB, Canada; ^5^St Vincent's Hospital Clinical School, University of New South Wales, Sydney, NSW, Australia; ^6^The University of Sydney Concord Clinical School, Faculty of Medicine and Health, Sydney, NSW, Australia

**Keywords:** cisplatin, TGF-β, DNA damage, DNA repair, follistatin, cell cycle, p21, p53

## Introduction

Lung cancer accounts for approximately 11% of all cancer cases, however the 5-year survival rate is often below 20%. Consequently, lung cancer is the leading cause of cancer related mortality worldwide (Bray et al., 2018). There are two major types of lung cancer; small cell lung cancer (SCLC), which accounts for ~15% of cases and non-small cell lung cancer (NSCLC), which accounts for ~85% (Herbst et al., 2018). NSCLC is further separated into lung adenocarcinoma (LUAD, ~50%), squamous cell carcinoma (~30%) and multiple smaller subtypes (~20%). Notably, up to 75% of NSCLC patients are diagnosed with advanced stage III/IV lung cancer (Walters et al., 2013), limiting surgical intervention.

While smoking is strongly associated with all lung cancer types, at least 20% of LUAD cases are from non–or never smokers (Herbst et al., 2018). Furthermore, while LUAD is characterized by a high somatic mutation rate, with deletion or mutation of TP53 occurring in up to 46% of cases, <20% of patients carry targetable mutations such as those within EGFR, ALK, or BRAF or NTRK (Arbour and Riely, 2019). Consequently, the overwhelming majority of LUAD patients receive platinum-based chemotherapy as standard of care.

Unfortunately, response rates to platinum in LUAD are below 30%, due to innate/acquired resistance and rate-limiting side-effects such as nephrotoxicity (Marini et al., 2018). Importantly, potential synergy between platinum chemotherapy and immunotherapy has emerged as a therapeutic opportunity in LUAD (Mathew et al., 2018). Therefore, improving platinum efficacy and identifying mechanism of resistance could significantly improve patient outcomes. In this opinion article, we cover several of the latest landmark publications that shed new light on the mechanisms of platinum resistance in LUAD.

## Overview of Platinum Chemotherapy

The anti-tumor abilities of cisplatin were identified over 50 years ago (Rosenberg et al., 1969). Since then platinum has become one of the most successful chemotherapeutics developed. It is essentially curative in testicular cancer, with survival rates >90% (Koster et al., 2013). It is also used with varying degrees of success to treat ovarian, head and neck, bladder and cervical cancer. Second and third generation cisplatin analogs have now been developed with the aim of lessening nephrotoxicity, neurotoxicity, ototoxicity, or providing better bioavailability and overcoming tumor resistance. Of these, carboplatin and oxiplatin are the most well-known, however nedaplatin, heptaplatin, lobaplatin and satraplatin are also used clinically (Wang and Lippard, 2005).

**Figure 1:**
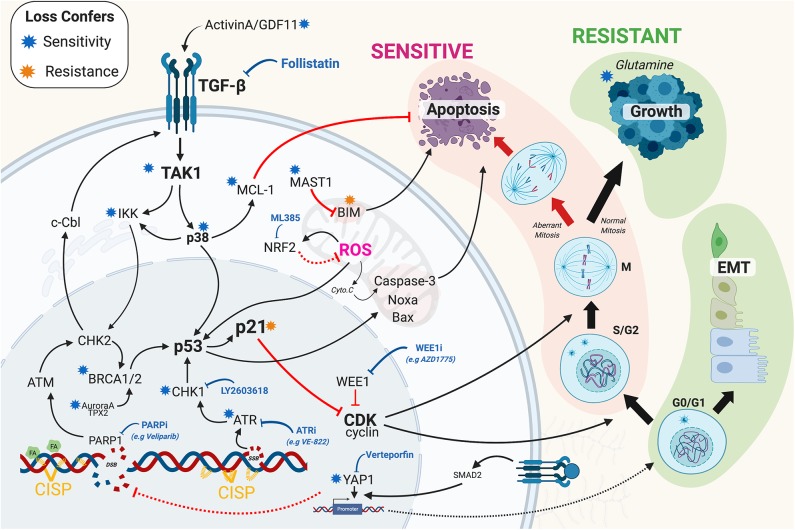
Overview of the recent mechanisms of resistance identified in lung adenocarcinoma. Briefly, mechanism of resistance work to increase repair of DNA damage and or prevent the triggering of cell death through inhibition of apoptotic signaling. Black solid arrows indicated activation. Red arrows indicate inhibition. Solid and dashed lines indicate direct and indirect mechanisms. Blue and orange stars indicate that loss (e.g., by RNAi, CRISPR, or chemical inhibition) confers sensitivity and resistance to cisplatin, respectively. Blue block arrow indicates potential therapeutic cisplatin synergistic treatment options. CISP, cisplatin; EMT, epithelial-mesenchymal transition; SSB/DSB, single/double strand breaks; FA, Fanconi Anemia pathway; ROS, reactive oxygen species; Cyto.C, Cytochrome C. The Figure created with BioRender.com.

Cisplatin and its derivates rely on their platinum group to exert killing. Platinum compounds can bind to many biological targets including DNA, RNA, and proteins (Stordal and Davey, 2007). The binding of cisplatin to DNA forms platinum-DNA adducts (Figure 1), which must be repaired by the cell. Approximately 90% of cisplatin-induced adducts are intra-strand crosslinks that are rapidly repaired mostly by the base-excision and nucleotide excision repair (BER, NER) pathways during G1 phase (Slyskova et al., 2018). In contrast, inter-strand crosslinks (ICL) represent <5% of cisplatin-induced adducts but are far more difficult for cells to remove as they are “hidden” within the DNA helix. ICLs prevent the unzipping of the double helix, creating a physical barrier to efficient DNA replication. The removal, largely by the Fanconi anemia (FA) pathway (Michl et al., 2016; Niraj et al., 2019; Smogorzewska, 2019), results in the formation of single and double strand breaks (SSBs and DSBs). The damaged DNA is then repaired by either the high-fidelity homologous recombination (HR) pathway during S/G2-phase (Karanam et al., 2012) or by the error-prone non-homologous end joining (NHEJ) pathway in G1 phase (Enoiu et al., 2012). The extent of, or failure to repair the DNA damage caused by cisplatin can result in cell death, accounting for the cytotoxic mode of action for most platinum agents. The exception is oxiplatin, which kill cells through increasing ribosome biogenesis stress (Bruno et al., 2017). For simplicity, here we will only focus on the mechanisms of cisplatin resistance in LUAD.

### Screening for Platinum Sensitisers

To date over 147 mechanisms of platinum resistance have been proposed (Stewart, 2007), yet there remains a lack of viable clinical options to improve response rates. To overcome this, several recent publications (Cheng et al., 2016; Jhuraney et al., 2016; Jin et al., 2018; Marini et al., 2018; Ding et al., 2019; Hsu C.-H et al., 2019), have looked at potential mechanisms of resistance in LUAD using unbiased screens, and detailed preclinical models. Based on these new data, it is clear that the main points of resistance arise from alterations to DNA repair, TGF-β signaling, cell cycle and apoptosis (Figure 1). Put simply, the ability of cisplatin to kill cells requires actively cycling cells that generate sufficient DNA damage and a functional apoptotic pathway to induce death. Consequently, disruption at any point along these pathways can prevent cell death, thereby reducing sensitivity to platinum mediated killing. Conversely, synergising therapies in general either block inhibitory cell death pathways, thereby lowering the threshold required to trigger death or increase the amount of damage induced by platinum.

### DNA Repair and Resistance to Platinum

The DNA Damage response (DDR), has been extensively reviewed (Jackson and Bartek, 2009; O'Connor, 2015; Pearl et al., 2015; Matt and Hofmann, 2016), as has its role in LUAD (O'Grady et al., 2014). Briefly, the DDR comprises of several functional layers including sensors (e.g., MRN complex, RPA, ATRIP), signaling kinases (e.g., ATM, ATR), damage mediators (e.g., 53BP1, BRCA1/2, H2AX), downstream kinases (e.g., CHK1/2), and cell cycle checkpoint effectors (e.g., p53, p21, WEE1). Unsurprisingly, defects at each level have been reported to regulate sensitivity to cisplatin in a variety of cancers including LUAD. Perhaps the best example of this is the well-reported link between BRCA1/2 mutations and sensitivity to cisplatin in breast cancer (Tutt et al., 2018). Similarly, the BRACness phenotype, which is defined as any defect that impacts HR repair and phenocopies the mutation or loss BRCA1/2 (Byrum et al., 2019b), is also strongly linked with sensitivity to platinum and PARP inhibitors (Ding et al., 2019), especially in ovarian (Pillay et al., 2019) and breast cancer (Tung and Garber, 2018). The links with BRCA1/2 mutations, BRACness and cisplatin sensitivity are less clear in LUAD. Although recent reports indicate that DNA methyltransferase inhibitors can induce a BRACness phenotype in NSCLC cells, sensitizing them to PARP inhibitors (Abbotts et al., 2019), and hence may extend to other DNA damage chemotherapies such as cisplatin (Figure 1). Early preclinical studies showed significant promise for directly inhibiting ATR kinase activity (Hall et al., 2014; Vendetti et al., 2015) to enhance cisplatin killing of LUAD cells. Interestingly, inhibition of ATM does not appear to synergise with cisplatin (Schmitt et al., 2017), although it may reduce the metastatic potential of cisplatin resistant LUAD cells (Shen et al., 2019). Furthermore, co-depletion of ATM and MCL-1 can re-sensitize cells to cisplatin (Zhang et al., 2017). While phase 1/2 trials of the CHK1 inhibitor LY2603618 in combination with cisplatin showed promising anti-tumor activity, but also caused significant thromboembolic side-effects (Wehler et al., 2017), indicating that despite promising results in SCLC (Sen et al., 2017; Hsu W.-H. et al., 2019; Nagel et al., 2019), Chk1 inhibitors may not translate to LUAD. Indirectly targeting the DDR has also shown some promise, with inhibition of the JMJD2 histone demethylase family re-sensitizing resistant LUAD to cisplatin by preventing ATR association to sites of DNA damage, thereby weakening the DDR (Duan et al., 2019). Similarly, targeting specific forms of the PP2A phosphatase complex (PPP2R2A2), which are responsible for dephosphorylating and inactivating ATM and ATR, enhanced sensitivity to PARP inhibition in LUAD by maintaining the DDR response (Kalev et al., 2012). What is becoming clear is that there are a number of non-canonical DDR pathways, many of which become upregulated during oncogenesis and can increase replication fork stability and counterbalance BRACness and BRCA mutations (Chaudhuri et al., 2016). A surprising recent example is the discovery that the mitotic kinase Aurora A and its targeting factor TPX2 can regulate 53BP1 and HR repair in a pathway parallel to BRCA1 (Byrum et al., 2019a), possibly explaining why shRNA knockdown of Aurora A sensitized LUAD cells to cisplatin (Cheng et al., 2016). These results highlight the need for additional research that maps all of the pathways regulating the DDR in LUAD.

### TGF-β Signaling, EMT and Resistance

The sensing and repair of cisplatin adducts does not happen in isolation from the rest of the cell or its local environment. The DDR signaling pathway is intimately integrated into multiple signaling networks, with a prime example being the transforming growth factor β (TGF-β) pathway. TGF-β regulates a multitude of cellular pathways including the DDR, cellular proliferation and the epithelial-mesenchymal transition (EMT). It plays both positive and negative roles in cancer development and progression. In established tumors, high TGF-β expression can drive metastasis, tumor heterogeneity and chemoresistance (Li J. et al., 2019). We recently demonstrated that members of the TGF-β pathway, including ACVR1B, TGFBR1, TAK1 and GDF11, mediated innate cisplatin resistance in LUAD (Figure 1), a possible consequence of epithelial airway cell lineage (Kretser et al., 2011). Critically, inhibition of activin receptor signaling reversed the resistance, as did blockage of activin A and GDF11 by the endogenous protein Follistatin (Marini et al., 2018). The mechanisms for TGF-β resistance are multifaceted, likely acting to suppress cell proliferation, apoptosis, and the DDR. In support, the antiapoptotic protein MCL-1 decreased upon chemical inhibition of the TGF-β pathway in cisplatin treated cells (Marini et al., 2018). TAK1 has also recently been shown to phosphorylate p38 MAPK and IKKα after DNA damage (Colomer et al., 2019), promoting ATM phosphorylation and increasing DNA repair, leading to chemoresistance. In turn, ATM can feedback into the TGF-β pathway, phosphorylating c-Cbl, stabilizing TβRII receptor and activating TFG-β signaling (Li Y. et al., 2019), creating a positive feedback loop (Figure 1). TGF-β can also drive EMT (Hao et al., 2019) and chemoresistance (Fischer et al., 2015), in part due increased YAP1 mediated transcription of TGF-β target genes (Pefani et al., 2016). Consequently, TGFBR1 and YAP1 inhibitors have been shown to be synergistic in GATA4 deficient (Hao et al., 2019) and EGFR-mutant (Cheng et al., 2016) lung cancers, respectively (Gao et al., 2019), offering another potential therapeutic approach to enhancing cisplatin selectivity.

### Cell Cycle and Apoptosis

In general, non-cycling cells are more resistant to cytotoxic chemotherapies such as cisplatin, however, proliferating cells that increase repair or reduce death signaling are more resistant, and often more deadly. Once a proliferating cell encounters DNA damage it must halt cell cycle progression so that repair can occur. If the damage is deemed too great, then apoptosis will be initiated, thereby preventing the damage being passed on to subsequent generations. The key central regulator of this decision pathway is p21^waf1/kip^, which inhibits G1 and G2 cell cycle progression (Burgess et al., 2019) and blocks caspase 3 dependent apoptosis (Suzuki et al., 2000). Interestingly, intermediate “goldilocks” levels of p21 strongly correlate with continued cell proliferation post cisplatin exposure, while low or high levels result in damaged cells undergoing senescence (Hsu C.-H et al., 2019). Similarly, over-riding the protective cell cycle checkpoints in S and G2 phase through WEE1 inhibition has also shown promise, especially in p53 null and mutant cell lines (Jhuraney et al., 2016; Richer et al., 2017). Interestingly, some resistant cycling cells become highly dependent on glutamine for a multitude of metabolic reactions. Consequently, removal of glutamine makes resistant cells highly sensitive to cisplatin, and lowers the threshold required to trigger apoptosis (Guidi and Longo, 2018). Similarly, metformin, which blocks glucose uptake and ATP production, has also been linked with increasing sensitivity to cisplatin (Liu et al., 2017; Riaz et al., 2019). While, inhibition of NRF2, which protects against hypoxia and reactive oxygen species (ROS), synergises with cisplatin by enhancing DNA damage (Singh et al., 2016; Shi et al., 2019). Notably, NRF2 is commonly upregulated in LUAD by KRAS (Tao et al., 2014) and mutant p53 (Tung et al., 2015). Disrupting apoptosis is another common mechanism, with upregulation of MAST1 in LUAD cells resulting in a rewiring of downstream MEK signaling and a reduction in pro-apoptotic protein Bim (Figure 1), thereby increasing the threshold required to trigger apoptosis (Jin et al., 2018). Likewise, mutations in SET containing 2 (SETD2), a histone methyltransferase, confers cisplatin resistance in LUAD by altering ERK signaling and inhibiting apoptosis (Kim et al., 2019). While, as mention, TAK1-p38 signaling results in an increase in anti-apoptotic MCL1 levels, raising the threshold required to trigger apoptosis (Marini et al., 2018).

## Discussion and Conclusion

A more complete understanding of the signaling, repair and apoptotic networks that are re-wired in LUAD will be key to improving platinum efficacy in LUAD. In addition, better temporal information on the dynamic nature of the signaling responses will greatly aid in the identification and prediction of resistance mechanisms. Any models will need to take into account cell cycle status, repair pathway and apoptotic thresholds in order to identify suitable synergising treatments. Finally, better preclinical models that more accurately model the dosing of platinum will be essential. Currently, the majority of studies rely on prolonged exposure, often >10-fold higher than what is achievable in patients (Urien and Lokiec, 2004; Jacobs et al., 2005). Screening of synergistic treatments using this extreme exposure may have increased the rate of false positives and failure of some preclinical studies to translate clinically. This is further cofounded by the disparate effect that platinum has on various organs (Yimit et al., 2019), especially the kidneys. Consequently, treatments such as Follistatin (Marini et al., 2018), which not only protect these vital organs but also enhance tumor selective killing, may have significant clinical potential. In summary, the advent of large-scale screens combined with detailed preclinical studies has given a greater understanding of the mechanisms of cisplatin resistance in LUAD, breathing new life into this stalwart of chemotherapy.

## Author Contributions

AG-R and JH co-wrote the initial draft. DW and DC co-wrote and edited the manuscript. AB conceived and wrote the article.

## Conflict of Interest

DW is a coinventor on a patent application relating to components of this work (U.S. 20180125936-A1). The remaining authors declare that the research was conducted in the absence of any commercial or financial relationships that could be construed as a potential conflict of interest.
